# Formability and Failure Mechanisms of Continuous Glass Fiber-Reinforced Polypropylene Composite Laminates in Thermoforming Below the Melting Temperature

**DOI:** 10.3390/polym16202885

**Published:** 2024-10-14

**Authors:** Qihui Ying, Zhixin Jia, Di Rong, Lijun Liu, Jiqiang Li

**Affiliations:** 1 School of Mechanical Engineering, Zhejiang University, Hangzhou 310027, China; 2 School of Mechatronics and Energy Engineering, NingboTech University, Ningbo 315100, China

**Keywords:** CGFRPP laminate, melting temperature, thermoforming properties, formability, failure mechanisms

## Abstract

In this study, the thermoforming formability of continuous glass fiber-reinforced polypropylene (CGFRPP) laminates below the melting temperature were investigated. The forming limits of CGFRPP laminates were explored using flexural tests, Erichsen tests and deep drawing tests. The failure mechanism of CGFRPP in thermoforming was investigated by observing typical failure specimens using a microscope. The results show that the flexural performance and Erichsen performance are optimal at 130 °C and 2 mm/min. At 160 °C and 100 mm/min, the deep drawing performance is optimal. The restriction of fibers by the matrix is affected by the deformation temperature, and the creation of defects is affected by the deformation rate. During forming, the CGFRPP laminates undergo shear and extrusion deformations, resulting in wrinkles, delamination, and fiber aggregation.

## 1. Introduction

Fiber-reinforced composites are used in aerospace and automotive applications as lightweight structural components due to their low density and high strength [1,2]. Continuous fiber-reinforced thermoplastic composites, such as CGFRPP composites, are commonly used in the manufacture of automotive parts, such as dashboards and door panels, because of their high strength, corrosion resistance, and ease of manufacture [3,4].

Laminates are the initial form of CGFRPP composites, which are formed into products via the integrated over-molding process [5,6]. Integrated over-molding is a process that combines hot press molding and injection molding. CGFRPP laminates are preheated, placed into molds, formed and injected into structural parts. Compared to conventional processes [7,8,9], integrated over-molding can produce structurally complex parts in a single pass.

Studying the forming properties of CGFRPP laminates is of great significance for product forming and process optimization [10,11]. Bigg et al. [12,13] found that semi-crystalline thermoplastic matrices can be permanently deformed between the crystallization temperature $T_{c}$ and the melting temperature $T_{m}$. Nishino [14] investigated the strain softening behavior of an epoxy resin matrix at elevated temperatures and found that the mechanical properties of the matrix polymer have a strong influence on the plastic deformation of carbon fiber-reinforced thermoset composites. There are no suitable standards for testing the formability of composite materials; thus, numerous scholars have conducted research on the use of metallic material standards. Analogous to studying the forming properties of metal sheets [15,16], the forming properties and failure mechanisms of composites can be investigated via flexural, deep drawing, and Erichsen tests. Uriya et al. [17,18,19,20] investigated the hot compression molding properties via bending, tensile, cupping, and flaring tests of UD-CF/EP composite sheets at elevated temperatures. The results showed that the tensile strength, flexural strength, and product structural form of the composites had a significant effect on their hot compression molding properties. Zheng et al. [21] investigated the molding properties of woven CF/PEEK sheets in a solid-state hot forming process. By determining the $T_{c}$ of PEEK and CF/PEEK, the forming properties as well as the failure mechanisms, were analyzed via flexural and Erichsen tests. Zhao et al. [22] experimented with a two-layer CGFRPP hemispherical hot stamping die, and the results showed that the suitable stamping depth should be less than 15 mm, the stamping speed should be less than 150 mm/min, and the preheating temperature should be about 200 °C.

However, there are fewer studies on the thermoforming of multilayered continuous fiber orthogonal layup thermoplastic composites. In this paper, the $T_{c}$ and $T_{m}$ of CGFRPP were tested via TGA and DSC. Then, the forming properties and forming limits of CGFRPP laminates in the glassy transition region were analyzed using flexural, Erichsen, and deep drawing tests. The failure mechanism of CGFRPP laminates in deformation was analyzed by observing typical failure specimens using EM and SEM.

## 2. Materials and Methods

### 2.1. Materials

The composite laminates (Kingply TM, supplied by the Guangzhou Jinfar Technology Company, Guangzhou, China) consisted of unidirectional continuous glass fibers and PP resin. The continuous fiberglass layup consists of four layers organized in a 0°/90°/90°/0° pattern, achieving a total thickness of 0.6 ± 0.1 mm. The heat deflection temperature of CGFRPP laminates was provided by the manufacturer as being 160 °C with a fiber mass fraction of 60 ± 2%. The test specimens were cut from laminates using precision engraving.

### 2.2. Thermal Analysis Tests

Thermogravimetric analysis (TGA) and differential scanning calorimetry (DSC) are commonly used thermal analysis techniques to investigate the changes in material properties during heating. The TGA and DSC specimens with uniform shapes and masses of 7–10 mg were placed in the holder. TGA tests were performed in a simultaneous thermal analyzer (TG209F1, NETZSCH, Selb, Germany) to investigate the thermal stability and compositional changes in CGFRPP. The temperature gradually increased from 20 to 700 °C at a rate of 10 °C/min under nitrogen protection, and the change in specimen weight was recorded by the sensor. DSC tests were performed in a differential scanning calorimeter (DSC25, TA Instruments, Shanghai, China) to investigate the $T_{c}$ and $T_{m}$ of CGFRPP. Under nitrogen protection, the specimen and the inert reference material were heated from 20 °C to 250 °C at rates of 5 °C/min, 10 °C/min, 15 °C/min, and 20 °C/min, respectively. The $T_{c}$ and $T_{m}$ of the specimen were obtained from the heat flow difference data recorded by the sensor. The degree of crystallinity $X_{c}$ can be calculated using Equation (1):

(1) $$X_{c}=\frac{∆H_{m}}{H_{f}\left(1-\alpha \right)}\times 100\%$$

where $∆H_{m}$ is the enthalpy of fusion of specimen at melting point $T_{m}$; $H_{f}$ is the enthalpy of melting when the material is fully crystallized, and the enthalpy of melting of fully crystallized polypropylene is 209 J/g. α is the mass fraction of glass fibers contained in the specimen.

### 2.3. Flexural Tests

**Figure 1:**
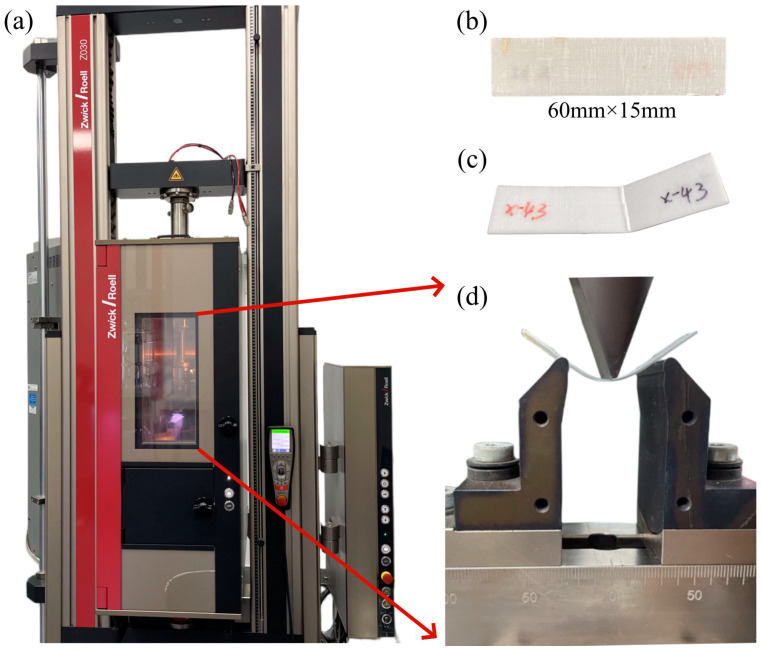
Flexural test at elevated temperature: (**a**) test equipment; (**b**) specimens; (**c**) failure specimen; (**d**) test mold.

**Figure 2:**
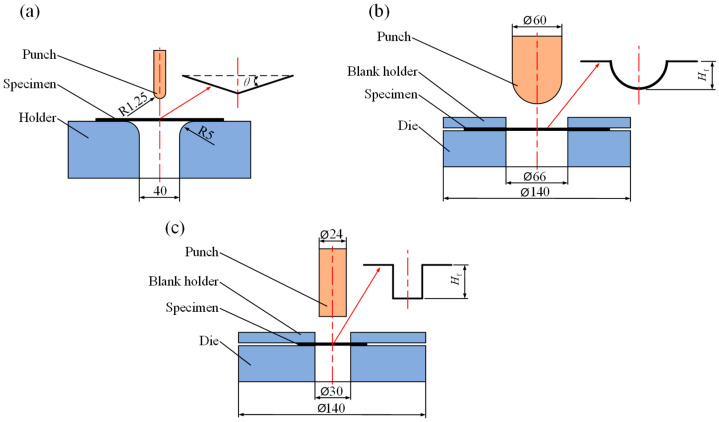
Schematic of thermoforming experiment: (**a**) flexural test; (**b**) Erichsen test; (**c**) deep drawing tests.

Flexural tests were conducted using an electric universal testing machine (Z030TE, Zwick/Roell, Ulm, Germany) with a span of 40 mm. The flexural test specimens were rectangular and had the following dimensions: 60 mm × 15 mm × 0.6 mm. The rectangular specimens were bent at temperatures ranging from 30 to 165 °C and at deformation rates from 2 to 100 mm/min. Five specimens were tested for each experimental condition to assess the flexural behavior and formability. Figure 1 shows the flexural test setup and specimens, and Figure 2a shows the schematic of the flexural test.

The flexural test specimens demonstrate both elastic and viscous behavior during deformation. To accurately represent the material’s actual behavior and minimize measurement errors caused by geometric effects, the flexural strength $\sigma_{b}$ and strains $\varepsilon_{b}$ are corrected using Equations (2) and (3).

(2) $$\sigma_{b}=\frac{3F_{b}L_{b}}{2bh^{2}}\left\{1+6{\left(\frac{S}{L_{b}}\right)}^{2}-3\left(\frac{Sh}{{L_{b}}^{2}}\right)\right\}$$

(3) $$\varepsilon_{b}=\frac{h}{L_{b}}\left\{6\frac{S}{L_{b}}-24.37{\left(\frac{S}{L_{b}}\right)}^{3}+62.17{\left(\frac{S}{L_{b}}\right)}^{5}\right\}$$

where $F_{b}$ is the maximum load, S is the deflection, and $L_{b}$ is the span; b and h represent the width and thickness of the specimens, respectively.

The modulus $E_{b}$ is calculated using Equation (4).

(4) $$E_{b}=\frac{\sigma_{b2}-\sigma_{b1}}{\varepsilon_{b2}-\varepsilon_{b1}}$$

The failure angle θ is used to characterize the flexural forming ability of the laminates, as shown in Equation (5).

(5) $$\theta =\arctan\left(\frac{2S}{L_{b}}\right)$$

### 2.4. Erichsen Tests

**Figure 3:**
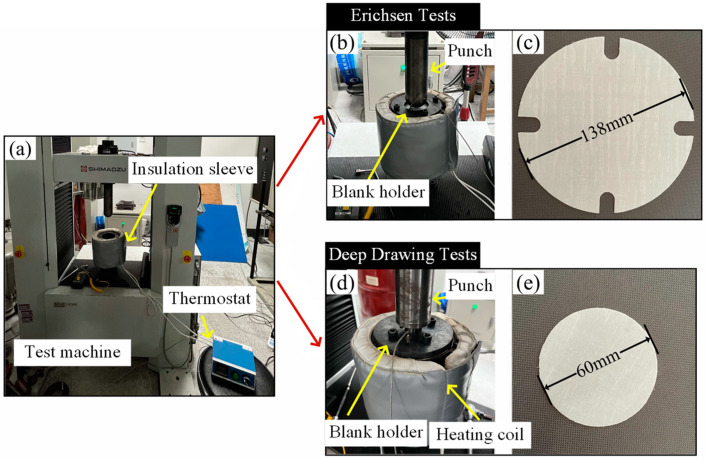
The Erichsen test and the deep drawing test at elevated temperatures: (**a**) test equipment; (**b**) Erichsen test mold; (**c**) Erichsen test specimens; (**d**) deep drawing test mold; (**e**) deep drawing test specimens.

The Erichsen test is a commonly used forming test that can be used to analyze forming capabilitues by studying the tensile deformation behavior of materials [20,23,24]. Although the Erichsen test is normally used in the study of metal sheets, some of the knowledge can be applied to composites. Erichsen tests were performed using a material high-temperature durability tester (AG-IC 100Kn, Shimadzu, Kyoto, Japan) to investigate the formability of CGFRPP laminates in thermoforming. The test conditions are shown in Table 1. The Erichsen test specimens were round with dimensions of ϕ 138 mm × 0.6 mm. The Erichsen test is shown in Figure 2b and Figure 3a–c.

In the Erichsen test, the CGFRPP laminate was positioned in the mold and secured using a blank holder. The heating coils heats the mold, blank holder, and specimen to a predetermined forming temperature. Then, the spherical punch made one stroke, and the hot stamping was complete. By recording the load *F* and displacement *H*, the limit Erichsen ratios LER were calculated using Equation (6):

(6) $$LER=\frac{H_{f}}{D_{Er}}$$

where $H_{f}$ is the displacement of the punch when the specimen was broken, and $D_{Er}$ is the diameter of the cup punch.

### 2.5. Deep Drawing Tests

The deep drawing test is used to evaluate the deformation capabilities of materials at elevated temperatures [19]. The deep drawing deformation capacity and forming limits of CGFRPP laminates in thermoforming were investigated. The equipment and experimental procedure of the deep drawing test are consistent with the Erichsen test. The deep drawing tests were performed on ϕ 60 mm × 0.6 mm round specimens according to the test conditions in Table 1. The deep drawing test is shown in Figure 2c and Figure 3a,d,e.

The limit deep drawing ratio LDR was calculated using Equation (7):

(7) $$LDR=\frac{H_{f}}{D_{Dr}}$$

where $H_{f}$ is the displacement of the punch when the specimen was broken, and $D_{Dr}$ is the diameter of the punch.

### 2.6. Microstructure Observation

The EM specimens and SEM specimens were sanded and polished, ultrasonically cleaned, and then dried at 60 °C for 1.5 h. The fracture cross-section was viewed using a super depth-of-field electron microscope (VHX-7000, KEYENCE, Osaka, Japan). The SEM specimens were coated with gold to improve surface conductivity prior to observation using a field emission scanning electron microscope (SU8010, HITACHI, Tokyo, Japan). It is reasonable to analyze the failure mechanisms by using the failed specimens [21,25].

## 3. Results and Discussion

### 3.1. Thermal Properties of CGFRPP Composites

**Figure 4:**
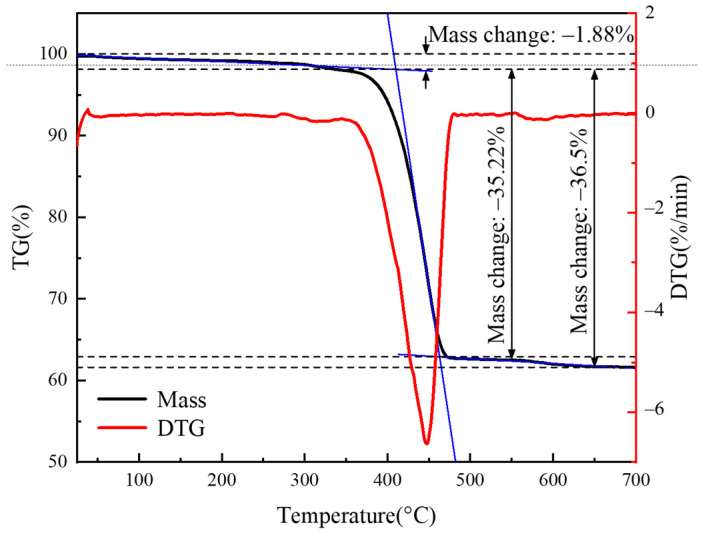
TGA results of CGFRPP composites.

Figure 4 shows the TGA results of the CGFRPP composites. At 410 °C, the mass of the CGFRPP laminate decreased by 1.88% due to volatilization. When the temperature increased to 460 °C, the mass of the laminate rapidly decreased by 35.22% due to the increased thermal decomposition of PP. When the temperature came to 700 °C, the decomposition of PP was complete, and the remaining undecomposed glass fibers eventually accounted for 61.62% of the total mass.

**Figure 5:**
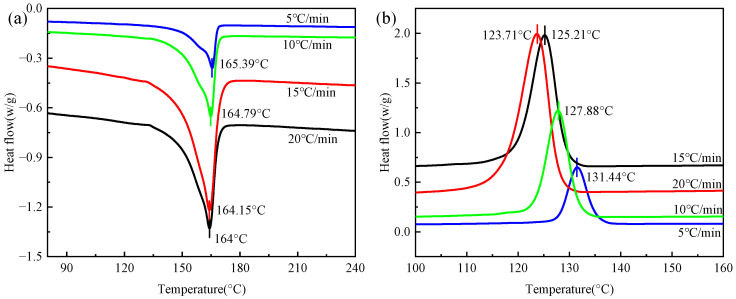
DSC results of CGFRPP composites: (**a**) warming phase; (**b**) cooling phase.

Figure 5 shows the DSC results of the CGFRPP composites. The $T_{m}$ and $T_{c}$ of CGFRPP are presented in Figure 5a and Figure 5b, respectively. At different rates of temperature change, the values of $T_{m}$ and $T_{c}$ were essentially similar; thus, the mean values were taken as being 165.58 °C and 127.06 °C, respectively. The $∆H_{m}$ at the $T_{m}$ of the laminate is presented in Table 2. Bringing $∆H_{m}$ and α into Equation (1), the $X_{c}$ of CGFRPP was calculated. The rate of temperature increase had a small fluctuating effect on crystallinity.

According to the results of existing research [12], semi-crystalline polymers are best formed between $T_{m}$ and $T_{c}$. Therefore, an experimental temperature range from 130 to 160 °C was selected.

### 3.2. Flexural Properties and Formability under Different Parameters

**Figure 6:**
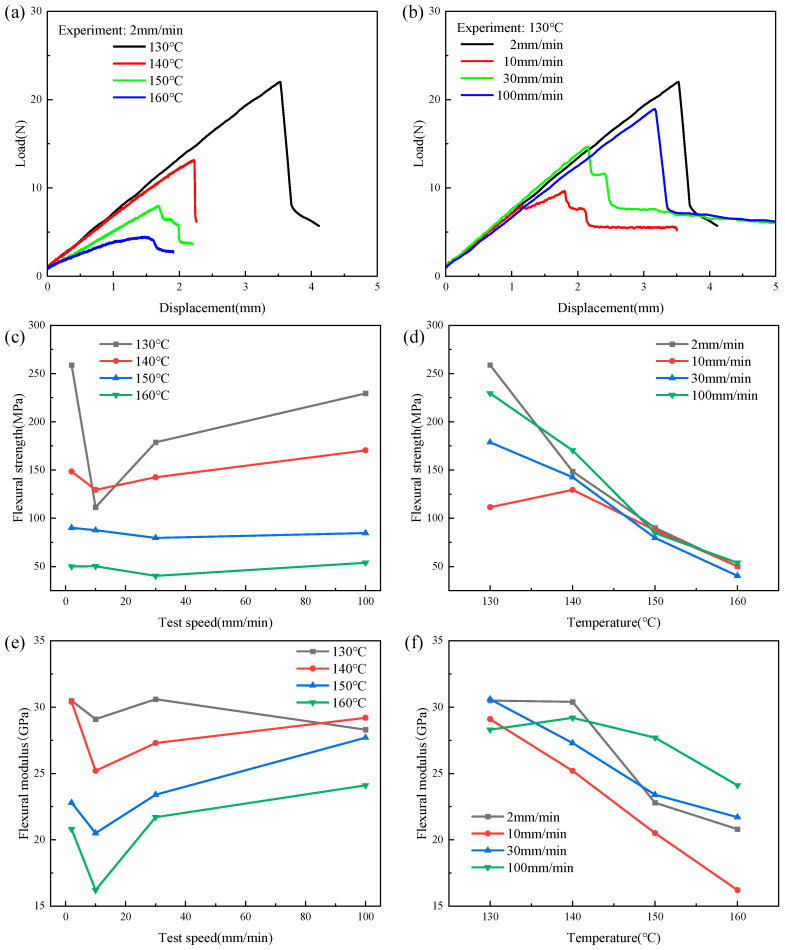
Results of the flexural test on CGFRPP composites: (**a**,**b**) load–displacement curve, (**c**,**d**) effect of speed or temperature on flexural strength, and (**e**,**f**) effect of speed or temperature on flexural modulus.

The flexural test results of the CGFRPP composites are shown in Figure 6. At the same testing speed, typical brittle fracture behavior appeared at temperatures of 130 °C and 140 °C, as illustrated in Figure 6a. The damage behavior transitions from a brittle fracture to viscoelastic deformation with a gradual increase in testing temperature. When the temperature is 150 °C and 160 °C, the deformation behavior of the laminate shows elastic and viscoelastic phases. As shown in Figure 6b, the specimen fails due to brittle fracture at lower or higher molding speeds. When the forming speed ranges from 10 mm/min to 30 mm/min, the specimen fails due to ductile fracture, which goes through the elastic stage, yielding stage, and strain softening stage. Therefore $\sigma_{b}$ and $\varepsilon_{b}$ are corrected using Equations (2) and (3) and $E_{b}$ is calculated. As shown in Figure 6c,e, $\sigma_{b}$ and $E_{b}$ fluctuate up and down with increasing speed at the same temperature. As the temperature nears the heat deflection temperature of CGFRPP, its viscoplastic behavior becomes more pronounced. The increased molecular motion at higher temperatures weakens the material’s rigidity and resistance to deformation, causing a significant reduction in both $\sigma_{b}$ and $E_{b}$. $\sigma_{b}$ reaches its maximum at 2 mm/min, and $E_{b}$ reaches its maximum at 30 mm/min. As shown in Figure 6d,f, both $\sigma_{b}$ and $E_{b}$ decrease substantially with increasing temperature at the same speed.

**Figure 7:**
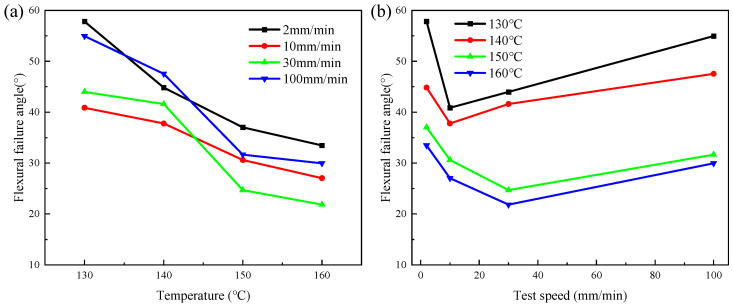
Flexural failure angle of CGFRPP composites under different molding conditions: (**a**) effect of deformation temperature on failure angle; (**b**) effect of deformation speed on failure angle.

Figure 7 shows θ of the CGFRPP composites under different molding conditions. The larger the value of θ, the better the flexural deformation property of the material. θ decreases as the temperature rises and shows a pattern of first decreasing and then increasing with rising speed, as shown in Figure 7a and Figure 7b, respectively. The CGFRPP laminate reaches a maximum flexural failure angle of 57.81 ° at a temperature of 130 °C and a speed of 2 mm/min.

**Figure 8:**
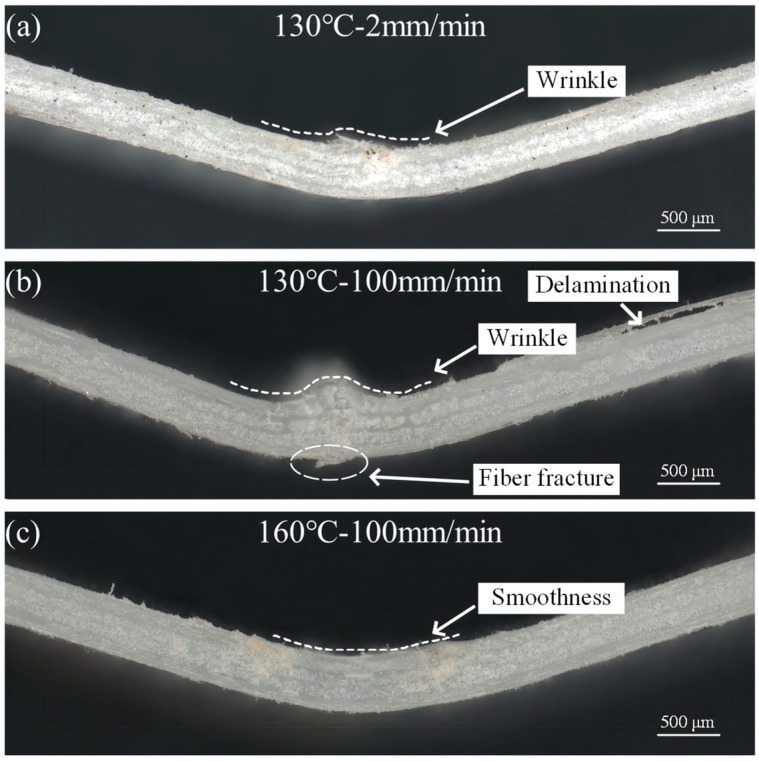
Typical failure specimen cross-section: (**a**) 130 °C-2 mm/min; (**b**) 130 °C-100 mm/min; (**c**) 160 °C-100 mm/min.

Figure 8 shows the typical failure cross-sections of the specimens. As shown in Figure 8a,b, at lower deformation temperatures and deformation speeds, the specimens showed small folds. As the deformation speed increases, the folds become larger, and the fracture exhibits fiber breaks along with interlayer delamination. As the deformation temperature reached the CGFRPP heat deflection temperature, the specimens showed no obvious failure, as shown in Figure 8c.

### 3.3. Erichsen Formability under Different Parameters

**Figure 9:**
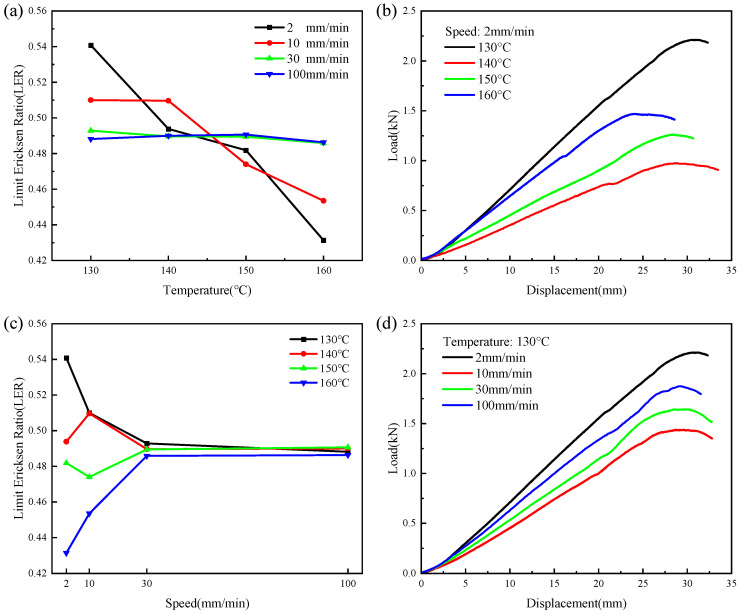
Results of the Erichsen test on CGFRPP composites: (**a**) effect of temperature on LER; (**b**) effect of displacement on force at different temperature; (**c**) effect of test speed on LER; (**d**) effect of displacement on force at different test speed.

Figure 9 shows the results of the Erichsen tests on the CGFRPP composites under different conditions. As shown in Figure 9a,c, in the range of deformation speed 2 mm/min-10 mm/min, LER decreases gradually with increasing temperature. In the range of deformation speed of 30 mm/min-100 mm/min, the fluctuation of LER was small. When the deformation speed is 2 mm/min and the deformation temperature is 130 °C, the LER reaches the maximum value of 0.54. The Erichsen formability of the CGFRPP laminate is shown in Figure 9b,d. The results show that the Erichsen formability of CGFRPP laminate is more affected by temperature at lower molding speeds and less affected at higher molding speeds.

**Figure 10:**
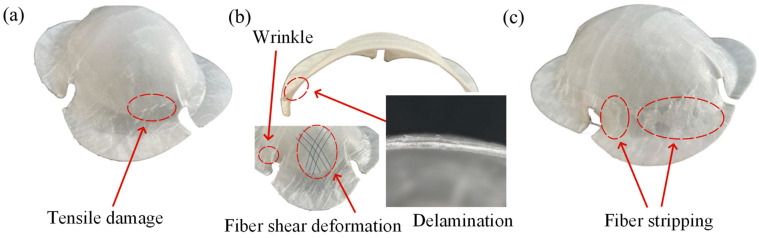
Typical fracture specimens of CGFRPP laminate in the Erichsen test: (**a**) 130 °C-2 mm/min; (**b**) 160 °C-20 mm/min; (**c**) 130 °C-100 mm/min.

Figure 10 shows typical forms of failure in the Erichsen test for a CGFRPP laminate. The main form of failure for the specimen shown in Figure 10a with a lower forming temperature is neck tensile failure. When the forming temperature increased to 160 °C, delamination occurred at the corner location, as exhibited by the specimen shown in Figure 10b. Since the forming temperature is close to the melting point of CGFRPP, the fibers are sheared under axial tensile stress and radial compressive stress. Comparing Figure 10a,c, the specimen with high forming speed has a large amount of fiber stripping.

### 3.4. Deep Drawing Properties and Formability under Different Parameters

**Figure 11:**
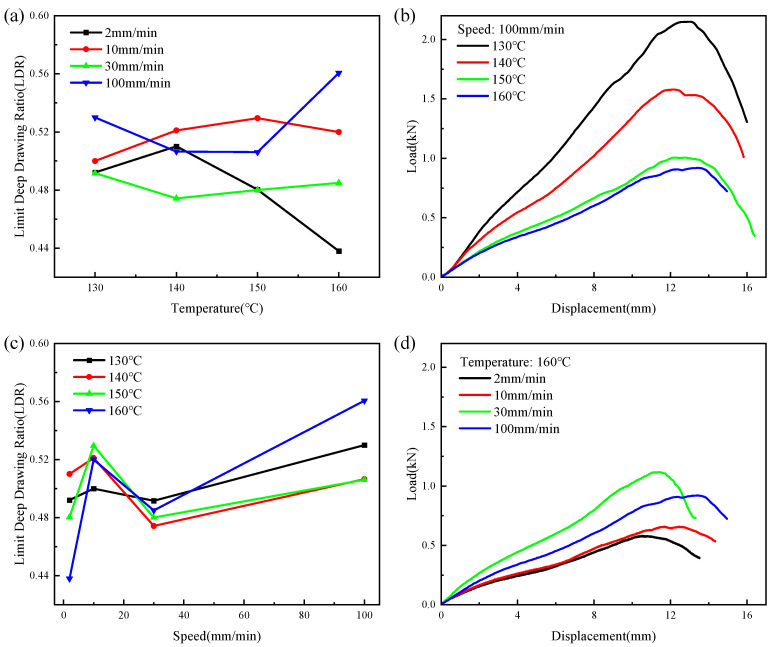
Results of the deep drawing test on CGFRPP composites: (**a**) effect of temperature on LDR; (**b**) effect of displacement on force at different temperatures; (**c**) effect of test speed on LDR; (**d**) effect of displacement on force at different test speed.

Figure 11 shows the results of the deep drawing test on the CGFRPP composites. The LDR fluctuates within a certain range for an increase in temperature when the speed is constant. When the temperature is constant, and with an increase in speed, the LDR shows a rise–drop–rise pattern. As shown in Figure 11b,d, the LDR reaches the maximum value of 0.56 at a deformation speed of 100 mm/min and a deformation temperature of 160 °C.

**Figure 12:**
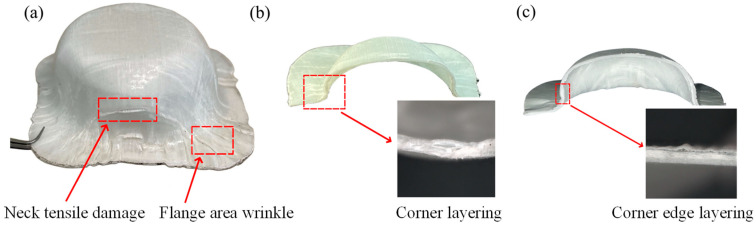
Typical fracture specimens of CGFRPP laminate in the deep drawing test: (**a**) 130 °C-100 mm/min; (**b**) 160 °C-100 mm/min; (**c**) 160 °C-2 mm/min.

Figure 12 shows typical forms of failure in the deep drawing test for a CGFRPP laminate. The main forms of the specimen shown in Figure 12a were neck tensile damage failure and fold defects at the flange. As the forming temperature increased to 160 °C, delamination occurred at the corner location of the specimen, as shown in Figure 12b. Comparing Figure 12b,c, the specimen with low forming speed has delamination failure near the corner, with wrinkle at the flange location.

### 3.5. Thermoforming of CGFRPP Laminate

**Figure 13:**
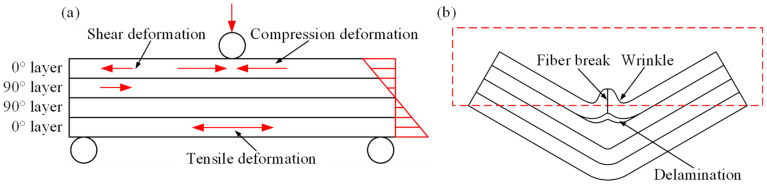
Schematic of flexural failure mechanism of CGFRPP laminates: (**a**) force schematic, and (**b**) failure schematic.

Figure 13 illustrates the damage mechanism of CGFRPP laminates during flexural tests. As shown in Figure 13a, during flexural forming, different internal stresses are generated in the internal layers of the specimen. Compressive deformation occurs in the upper layer and tensile deformation in the lower layer. The layers are connected by resin, and the middle layer is subjected to opposite forces, resulting in shear deformation. The wrinkle is caused by the upper material toward the center. In deformation, the resin layer between layers gradually expands, and fiber breakage and delamination will occur, as shown in Figure 13b.

When the forming temperature is 130–140 °C, the PP is less tough and the bonding of the fibers to the matrix is large. When subjected to external forces, the material is able to maintain good elastic deformation behavior. When the load reaches its bearing limit, debonding occurs at the interface between the PP matrix and the glass fibers. Cracks form and expand rapidly, and the fibers break. When the forming temperature is between 150 °C and 160 °C, the increase in ambient temperature softens the PP matrix and increases the ductility of the matrix, while weakening the interaction of fiber/matrix [26,27]. As a result, the layers are susceptible to interlayer slip, leading to resin layer expansion and interlayer delamination.

With a forming speed of 2 mm/min to 10 mm/min, the force loading is slow, and the PP matrix and glass fibers have more time to respond to the external load. As the strain rate increases, more regions will be involved in the deformation, which can easily lead to damage [28,29]. The effect of forming speed on the flexural properties of the composites decreases when the temperature is 160 °C, as well as the strength and modulus of the composites are low.

**Figure 14:**
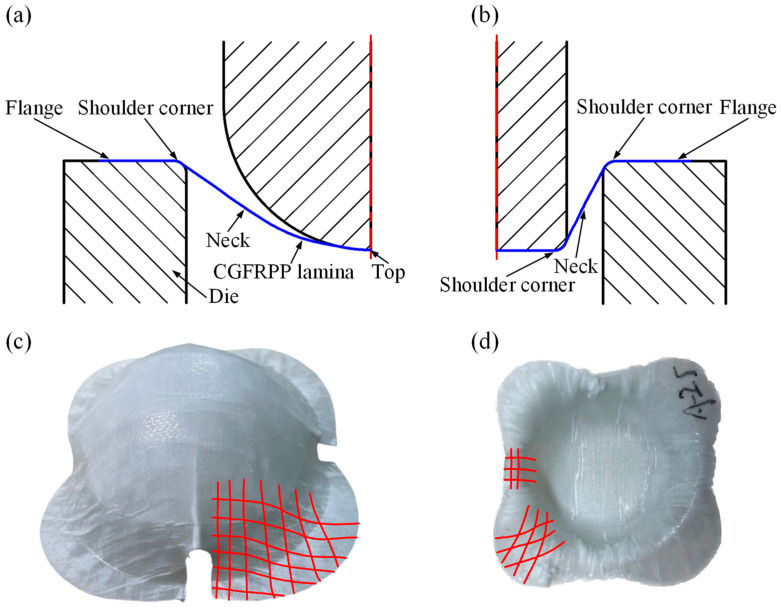
Thermoforming test results of CGFRPP laminate: (**a**) schematic of the Erichsen test; (**b**) schematic of the deep drawing test; (**c**) deformation of the Erichsen specimen; (**d**) deformation of the deep drawing specimens.

Figure 14 is the thermoforming test results of the CGFRPP laminate. As shown in Figure 14a,b, the center of the specimen conforms to the punch during thermoforming, causing the shoulder to bend and stretch. As the amount of deformation increases, the material at the top is stretched while the material at the corners undergoes in-plane extrusion deformation [21]. As shown in Figure 14c,d, after the deformation is completed, the internal continuous fiber orientation is changed [29]. Due to the in-plane compressive deformation of the material during the forming process, a significant amount of matrix and fibers in the specimen are continuously extruded and aggregate at the corners. This results in the extrusion of otherwise orthogonal fibers, leading to the formation of wrinkles [30].

When the deformation temperature is low, the viscous fluidity of the PP matrix is limited, restricting the material’s ability to deform plastically. As a result, tensile damage occurs at the top of the specimen, while flexural damage is observed at the shoulder during molding. As the deformation temperature gradually increases, the matrix strength and fiber/matrix interaction weaken. This leads to shear deformation of the fibers, with the shoulder fibers fracturing first and significant folding occurring at the flange. In the deep drawing test, the punch exerted intense stretching on the shoulder of the specimen. At higher deformation speeds, the base material exhibited higher tensile strength, which allowed the material to withstand greater deformation prior to crack formation. When the deformation speed is low, microscopic defects present in the material such as porosity and fiber enrichment are amplified during deformation, leading to earlier crack formation. In the Erichsen test, the sphere punch can load the force uniformly on the specimen, and the slow deformation speed favors the deformation of the composite [31]. Therefore, the maximum LER and LDR of CGFRPP appeared with different deformation speeds.

**Figure 15:**
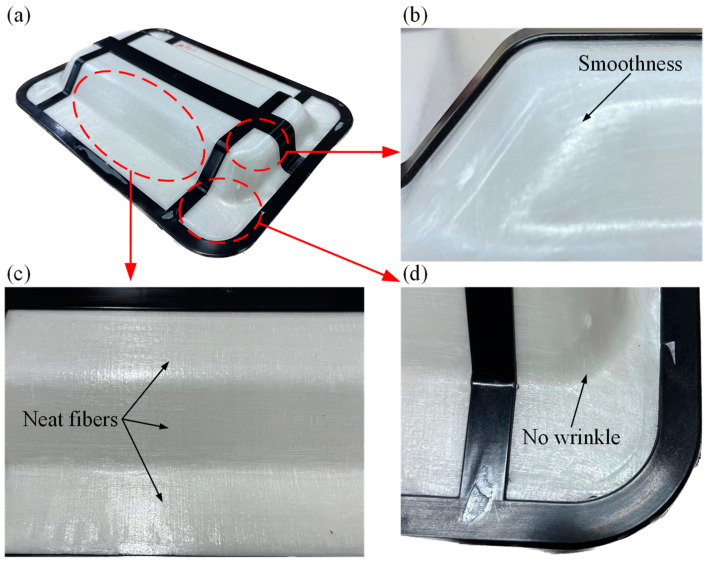
Validation of thermoforming skeleton for integrated over-molding: (**a**) composite product; (**b**) large rounded corner area; (**c**) flat area; (**d**) small rounded area.

Figure 15 is the validation of thermoforming skeleton for integrated over-molding. As shown in Figure 15a, the white portion of the product is thermoformed CGFRPP with dimensions conforming to LER and LDR. By increasing the fillet radius, the CGFRPP surface is free of visible defects. With the addition of slope, the fiber orientation at the flange, shoulder and top remains essentially the same with no wrinkle, as shown in Figure 15b–d.

## 4. Conclusions

The thermophysical properties and thermoforming properties of CGFRPP prepregs were investigated using different experimental methods, and the thermoforming failure mechanisms were analyzed. The main conclusions can be drawn.

(1) CGFRPP laminates have good thermal stability, and the quality starts to change drastically at 410 °C, which is in line with the thermal deformation condition. The optimum molding temperature range for CGFRPP laminates is 130–160 °C, and the crystallinity is 54.26%.
(2) The forming limits of CGFRPP laminates at temperatures below the melting temperature were investigated using flexural tests, Erichsen tests, and deep drawing tests. The thermoforming properties of the CGFRPP laminates are affected by the deformation speed and deformation temperature. The optimal flexural properties and LER of the CGFRPP laminates occur at a deformation temperature of 130 °C and a speed of 2 mm/min. In contrast, the highest LDR is achieved at a deformation temperature of 160 °C and a speed of 100 mm/min.
(3) In the deformation process, plastic deformation, shear deformation, and compression will occur in each part of CGFRPP laminates. Wrinkles and delamination tend to form at the corners and flanges. The apparent quality of CGFRPP thermoformed parts can be optimized by increasing the corner radius and adding slope.

## Figures and Tables

**Table 1:** Deep drawing and Erichsen test conditions.

Experiment	Value	Unit
Temperature	130, 140, 150, 160	°C
Speed	2, 10, 30, 100	mm/min
Blanking Torque	10	N·m

**Table 2:** Crystallization properties at different heating rates.

β/°C·min^−1^	$T_{m}$/°C	$T_{c}$/°C	$∆H_{m}$/J·g^−1^	$X_{c}$/%
5	165.39	131.44	38.76	48.32
10	164.79	127.88	41.04	51.16
15	164.15	125.21	37.64	46.92
20	164.00	123.71	39.87	49.70
Material values	164.58	127.06	39.33	49.03

## Data Availability

The original contributions presented in the study are included in the article, further inquiries can be directed to the corresponding author.
